# Evaluation of the right atrial phasic functions in patients with anterior ST-elevation myocardial infarction: a 2D speckle-tracking echocardiography study

**DOI:** 10.1186/s12872-022-02546-4

**Published:** 2022-03-14

**Authors:** Mokhtar Eisvand, Reza Mohseni-Badalabadi, Ali Hosseinsabet

**Affiliations:** Cardiology Department, Tehran Heart Center, Tehran University of Medical Sciences, Karegar Shomali Street, Tehran, Islamic Republic of Iran

**Keywords:** Right atrium, Myocardial infarction, Speckle-tracking echocardiography, Coronary artery disease

## Abstract

**Background:**

Evidence suggests that changes in left ventricular systolic and diastolic functions may affect right atrial (RA) phasic functions. We aimed to evaluate RA phasic functions in the presence of anterior ST-elevation myocardial infarction (ASTEMI) as an acute event and to compare the findings with those in a control group.

**Methods:**

We recruited 92 consecutive ASTEMI patients without accompanying significant stenosis in the proximal and middle parts of the right coronary artery and 31 control subjects, matched for age, sex, diabetes, and hypertension. RA phasic functions were evaluated concerning their longitudinal 2D speckle-tracking echocardiography-derived markers. The ASTEMI group was followed up for all-cause mortality or reinfarction.

**Results:**

In the ASTEMI group, RA strain was reduced during the reservoir (33.2% ± 4.3% vs 30.5% ± 8.1%; *P* = 0.021) and conduit (16% [12–18%] vs 14% [9–17%]; *P* = 0.048) phases. The other longitudinal 2D speckle-tracking echocardiography-derived markers of RA phasic functions were not different between the 2 groups. RA strain and strain rate during the contraction phase were predictive of all-cause mortality or reinfarction (hazard ratio = 0.80; *P* = 0.024 and hazard ratio = 0.39; *P* = 0.026, respectively).

**Conclusions:**

Based on 2D speckle-tracking echocardiography, in the ASTEMI group, compared with the control group, RA reservoir and conduit functions were reduced, while RA contraction function was preserved. RA contraction function was predictive of all-cause mortality or reinfarction during the follow-up period.

**Supplementary Information:**

The online version contains supplementary material available at 10.1186/s12872-022-02546-4.

## Introduction

The right atrium (RA) is the first cardiac chamber to receive blood from the peripheral arteries. The phasic functions of the RA modulate the function of the right ventricle (RV) through the storage of blood in the ventricular systole (RA reservoir function), the conveyance of blood to the ventricle during the early diastole (RA conduit function), and the delivery of the blood left in the RA by late diastolic contractions (RA contraction function) [1]. Consequently, in the long term, RA phasic functions may affect left ventricular (LV) function and cardiac output. On the other hand, the diastolic impairment of the LV can result in the diastolic impairment of the RV [2]. Additionally, RA pressure is correlated with LV diastolic pressure [3]. Hence, in the presence of LV diastolic dysfunction, the RA is exposed to increased RV diastolic pressure, which may affect RA phasic functions [4]. It is reasonable that in the backward route, acute changes in LV function induced by myocardial infarction (MI) may affect RA phasic functions. The phasic functions of the RA are independently associated with major adverse cardiac events in patients with acute MI [5]. RA phasic functions are impaired in the presence of inferior MI and RVMI compared with the presence of inferior MI in the absence of RVMI; nonetheless, the effects of anterior LVMI have yet to be clarified [6, 7].

In the recent decade, 2D speckle-tracking echocardiography (2DSTE) has been used by researchers to assess RA phasic functions extensively. Indeed, 2DSTE has become one of the main methods to evaluate RA phasic functions, with investigators having reached a consensus regarding how to measure RA phasic functions in terms of deformation markers [8, 9]. This method is capable of demonstrating the effects of hypertension, diabetes, autoimmune diseases, and RVMI on the phasic functions of the RA [6, 10–12]. Further, longitudinal 2DSTE-derived deformation indices are associated with RA-related hemodynamic measurements [13]. Other advantages of 2DSTE are its reproducibility and feasibility in the evaluation of RA phasic functions [14, 15]. In addition, longitudinal 2DSTE-derived deformation markers are correlated with cardiovascular magnetic resonance myocardial feature tracking-derived deformation markers [16].

The RA works in interaction with the RV, and its phasic functions are to some extent but not completely dependent on RV function as is the case in the relationship between left atrial (LA) function and LV function [17, 18]. All cardiac chambers are interconnected, and structural or hemodynamic changes in 1 chamber may impact the other chambers such as the effects of LV remodeling on RA phasic functions [6, 19].

In this study, hypothesizing that anterior ST-elevation myocardial infarction (ASTEMI) could affect RA phasic functions, we aimed to evaluate the phasic functions of the RA as assessed by 2DSTE in the presence of ASTEMI (the culprit lesion in the left anterior descending artery) and to compare the findings with those in a control group.

## Methods

### Study population

Between September 2019 and February 2021, we recruited patients with ASTEMI who had undergone successful primary percutaneous coronary interventions and were in the sinus rhythm. The fourth universal definition of MI was considered for the diagnosis of ASTEMI [20]. The exclusion criteria were composed of a history of atrial fibrillation, the presence of 50% or more than 50% stenosis in the proximal and middle parts of the right coronary artery, a history of uncontrolled thyroid diseases, autoimmune diseases, previous percutaneous coronary interventions, cardiac surgeries, pacemaker implantation, bundle branch blocks, any degree of valvular stenosis, moderate or more-than-moderate valvular regurgitation, a history of cardiomyopathies, a previous MI, liver diseases, creatinine levels exceeding 1.5 mg/dL, hemodynamic instability in first 3 days after hospital admission, and a neglected MI. Ultimately, 92 patients with ASTEMI were included in our study. The patients’ drug history at admission was taken, and venous samples for laboratory examinations were drawn on the first morning after admission. The patients were treated in keeping with the latest validated recommendations [21–23].

The clinical endpoint was considered to be a composite of reinfarction or all-cause mortality that presented as major adverse cardiovascular events (MACE). The patients were followed up after index ASTEMI up to the end of January 2022 by telephone calls or outpatient visits, and medical documents were evaluated for the confirmation of reinfarction or death.

The control group, selected from a pool of subjects who had undergone elective coronary angiography between July and February 2019, consisted of 31 subjects who were matched with the case group in terms of age, sex, hypertension, and diabetes. The inclusion criteria for the control group were the absence of significant coronary artery stenosis (50% or > 50%) and the sinus rhythm. The exclusion criteria were the same as those for the ASTEMI group. Laboratory examinations for the control group were performed between pre-admission days 0 and 10.

Hypertension was defined as the consumption of antihypertensive drugs or a history of blood pressure exceeding 140/90 mm Hg in 2 separate measurements. Diabetes was defined as the use of antidiabetic agents or insulin or HbA1c levels exceeding 6.4% or fasting blood glucose levels exceeding 126 mg/dL in 2 isolated samples.

The study protocol was approved by our institutional review board, and written consent was obtained from the participants.

### Standard echocardiography

Echocardiography was performed during the first 72 h after hospital admission for the patients with ASTEMI and on the discharge day for the control group. The echocardiographic examinations were performed while the patients were in the left lateral decubitus position and monitored by 1-lead electrocardiography. A commercial setting (Philips, Affinity 70C, Andover, MA, USA), with an S5-1 probe, was applied. All the echocardiographic evaluations were done by a single cardiologist highly specialized in echocardiography. LV volume at end-systole and end-diastole was measured in apical 4- and 2-chamber views, and LV ejection fraction was calculated according to the modified Simpson method. Mid-ventricular RV diameter at end-diastole, RV end-diastolic and end-systolic areas and fractional area change in the RV-focused 4-chamber view, and tricuspid annular plane systolic excursion with the M-mode in the standard apical 4-chamber view were obtained. The tricuspid flow was obtained by pulsed-wave Doppler from the modified apical 4-chamber view, and peak early diastolic and late diastolic flows (E and A, respectively) and the deceleration time of the E wave were measured. The peak pressure gradient of the tricuspid regurgitant flow, if obtainable, was measured. By pulsed-wave tissue Doppler imaging, the systolic, early diastolic, and late diastolic waves of the lateral tricuspid were recorded, and the peak of these waves was measured. We followed the recommendations of the American Society of Echocardiography for standard echocardiography measurement [24, 25].

### 2DSTE

**Fig. 1:**
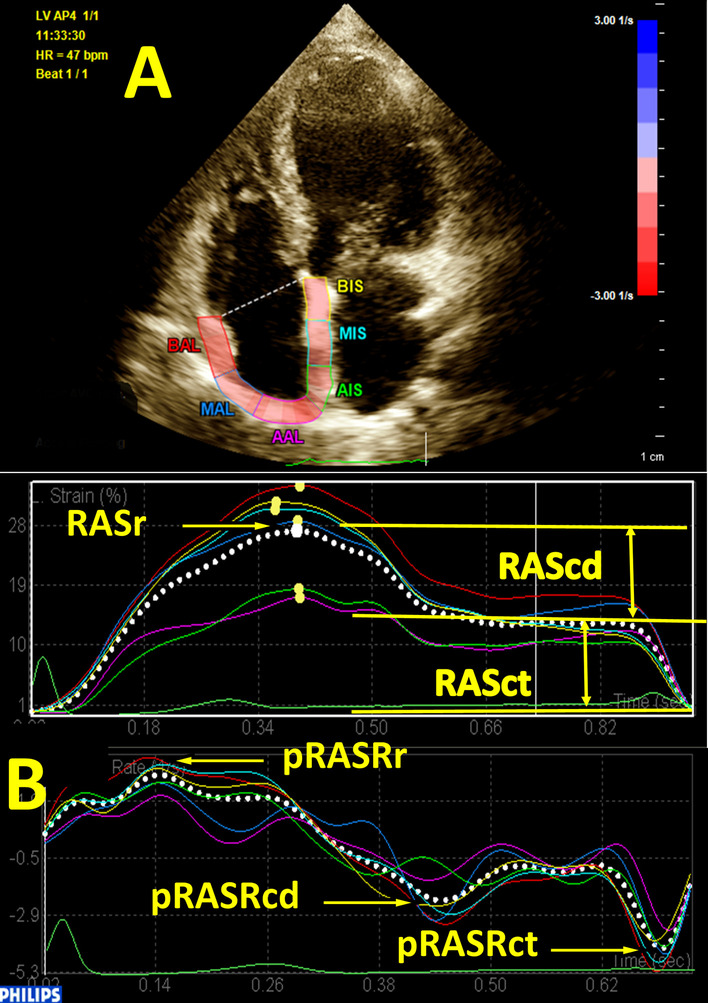
The image depicts 2D speckle-tracking echocardiography of the right atrium in the modified apical 4-chamber view. **A** strain curves, **B** strain rate curves. pRASRcd, peak right atrial longitudinal strain rate during the conduit phase; *pRASRct* peak right atrial longitudinal strain rate during the contraction phase, *pRASRr* peak right atrial longitudinal strain rate during the reservoir phase, *RAScd* right atrial longitudinal strain during the conduit phase, *RASct* right atrial longitudinal strain during the contraction phase, *RASr* right atrial longitudinal strain during the reservoir phase

Three consecutive cardiac cycles at end-expiration from the apical RV-focused 4-chamber view were obtained by the same cardiologist for 2DSTE at a rate of 47 ± 5 frames per second. The 2DSTE evaluations were done using QLAB 13 with the aCMQ package. At end-diastole, via the 3-click method, the medial and lateral tricuspid annuli and the mid-roof of the RA were defined. Thereafter, the endocardial and epicardial layers of the RA were traced automatically by the software, and the RA was divided into 6 equal segments. If required, appropriate adjustments were made with respect to the traced line for the endocardial and epicardial borders of the RA. Subsequently, by the selection of the “Compute” button, global strain and strain rate curves were visualized while the peak R was considered the 0 level. The strain curve has a peak in the systole, a plateau in the early diastole, and a nadir in the late diastole. The difference between the systolic peak and the late diastolic nadir was considered RA strain during the reservoir phase (RASr), the difference between the systolic peak and the early diastole was considered RA strain during the conduit phase (RAScd), and the difference between the early diastolic peak and the late diastolic nadir was considered RA strain during the contraction phase (RASct). The strain rate curve has 1 positive systolic peak (pRASRr, a marker of the reservoir function), 1 negative early diastolic peak (pRASRcd, a marker of the conduit function), and 1 late diastolic negative peak (pRASRct, a marker of the contraction function). The same method was applied for 2DSTE on the RV, and the 3-click points were the medial and lateral tricuspid annuli in the RV and its apex. The systolic strain curve of the RV has 1 systolic peak as a marker of the systolic function. The strain rate of the RV has 1 negative systolic peak as a marker of the systolic function and 2 positive peaks in early and late diastole as the indices of the diastolic function in the early and late diastole, respectively (Fig. 1). These deformation indices were measured for 3 segments of the RV free wall, and their average was considered the global value. The aforementioned deformation indices for the RA and the RV were measured in 3 cardiac cycles, and their mean was presented. We followed the recommendations of the American Society of Echocardiography in this field [9].

The aCMQ package depicted changes in the RA volume during the cardiac cycle, enabling the measurement of maximal RA volume, minimal RA volume, and pre–P-wave RA volume. The difference between maximal and minimal RA volumes was considered the total emptying volume (TEV), the difference between maximal and pre–P-wave volumes was considered the passive emptying volume (PEV), and the difference between pre–P-wave and the minimal RA volumes was considered the active emptying volume (AEV). The volumetric parameters of RA phasic functions were calculated as follows:

Reservoir function:

- RA total emptying fraction = 100 × TEV/maximal RA volume
- Expansion index = 100 × TEV/minimal RA volume

Conduit function:

- Passive emptying fraction = 100 × PEV/maximal RA volume
- Passive emptying percent of total emptying = 100 × PEV/TEV

Contraction function:

- Active emptying fraction = 100 × AEV/pre-P RA volume
- Booster active emptying percent of total emptying = 100 × AEV/TEV

Twenty-seven subjects (22%) were randomly selected for intra-and interobserver variability analysis. The first cardiologist and another cardiologist, who was also highly qualified in 2DSTE, independently measured the deformation markers of RA phasic functions 3 months after the study was completed.

### Statistical analysis

Categorical data were shown as frequencies and percentages and compared using the χ^2^ or Fisher exact test, whichever one was appropriate. Continuous data were demonstrated as the mean and the standard deviation if they were normally distributed and as the median and the interquartile range if they were not normally distributed. The continuous data were compared using the *t* test if they were normally distributed and otherwise compared using the Mann–Whitney *U* test. Inter-and intraobserver variabilities were analyzed using intraclass correlation coefficients. A *P* value of less than 0.05 was considered significant. Univariate Cox linear regression was applied to calculate the hazard ratio (HR) and its 95% confidence interval (CI) for the 2DSTE-derived indices of RA phasic functions. The statistical analyses were performed with IBM SPSS Statistics for Windows, version 24 (Armonk, NY: IBM Corp).

## Results

The study population’s demographic characteristics, as well as clinical and laboratory data, are presented in Table 1 in brief and Additional file 1: Table S1 in detail. The mean heart rate was higher in the ASTEMI group (*P* < 0.001), as were the mean levels of fasting blood sugar and low-density lipoprotein (*P* = 0.014 and *P* < 0.001, respectively). The patients in the ASTEMI group smoked more cigarettes (*P* = 0.006) and consumed fewer beta-blockers (*P* < 0.001), statins (*P* ≤ 0.001), and aspirin (*P* < 0.001) at admission according to their drug history than the control subjects. In the ASTEMI group, significant stenosis in the left anterior descending artery was detected in all the patients, in the left circumflex artery in 15 (16%), in the distal portion of the right coronary artery in 9 (10%), and in the left main in 2 (2%). Seventy patients with ASTEMI (76%) had single-vessel disease, 19 (21%) had double-vessel disease, and 3 (3%) had triple-vessel disease. The time interval between symptom presentation and echocardiography was 26.75 (21.0–38.0) hours, the time interval between admission and echocardiography was 21.25 (15.0–31.5) hours, and the time interval between revascularization and echocardiography was 20.25 (14.0–30.75) hours.

**Table 1:** Demographic, clinical, and biochemical characteristics of the study groups

Characteristics	Control group (n = 31)	ASTEMI group (n = 92)	*P* value
Sex (male) (%)	27 (87)	80 (87)	> 0.999
Age (years)	55 ± 11	56 ± 11	0.636
Body mass index (kg/m^2^)	28.7 ± 3.7	27.8 ± 4.8	0.354
Diabetes (%)	9 (29)	27 (29)	0.973
Hypertension (%)	11 (36)	32 (35)	0.944
Cigarette smoking (%)	7 (23)	47 (51)	0.006
Family history of coronary artery disease (%)	6 (19)	21 (23)	0.686
Aspirin (%)	19 (61)	12 (13)	< 0.001
Statins (%)	17 (55)	11 (12)	< 0.001
Beta-blocker use (%)	13 (42)	7 (8)	< 0.001
Fasting blood sugar (mg/dL)	101 (96–136)	117 (104–160)	0.014
Serum low-density lipoprotein level (mg/dL)	81 (56–97)	106 (86–124)	< 0.001

*ASTEMI* anterior ST-elevation myocardial infarction

The standard echocardiography data are presented in Table 2 in brief and Additional file 1: Table S2 in detail. LV ejection fraction was reduced in the ASTEMI group by comparison with the control group (49% ± 9% vs 59% ± 6%; *P* < 0.001). LV end-systolic volume index was increased in the ASTEMI group (*P* < 0.001). In the patients with ASTEMI, RV end-diastolic and systolic areas were decreased (*P* = 0.001 and *P* < 0.001, respectively) and RV fractional area change was increased (*P* = 0.001). In the ASTEMI group, the deceleration time of the E wave and the e′/a′ ratio were decreased (*P* = 0.021 and *P* = 0.024, respectively), and a′ was increased (*P* < 0.001). Maximal, minimal, and pre-P RA volume indices were lower in the patients with ASTEMI than in the control group (*P* < 0.05). However, the volumetric parameters of RA phasic functions were not different between the 2 study groups.

**Table 2:** Standard echocardiography data of the study groups

Variables	Control group (n = 31)	ASTEMI group (n = 92)	*P* value
Heart rate (bpm)	62 ± 9	79 ± 13	< 0.001
Systolic blood pressure (mm Hg)	126 ± 15	120 ± 17	0.117
Diastolic blood pressure (mm Hg)	80 ± 7	78 ± 11	0.310
LVEDV index (mL/m^2^)	49 ± 9	50 ± 11	0.542
LVESV index (mL/m^2^)	20 ± 4	29 ± 8	< 0.001
LVEF (%)	59 ± 6	42 ± 7	< 0.001
RVEDA (cm^2^)	19.7 ± 3.3	16.5 ± 4.6	0.001
RVESA (cm^2^)	10.8 ± 2.2	8.1 ± 2.9	< 0.001
RVFAC (%)	45 ± 7	51 ± 9	0.001
RA volumetric parameters			
Maximum RA volume index (mL/m^2^)	20 (17–27)	17 (13–22)	< 0.001
Minimum RA volume index (mL/m^2^)	9 (7–13)	7 (6–10)	0.005
Pre-P RA volume index (mL/m^2^)	15 (13–21)	12 (10–15)	< 0.001
Total emptying volume (mL)	21 (20–29)	17 (12–23)	0.001
Passive emptying volume (mL)	11 (9–14)	8 (5–13)	0.005
Active emptying volume (mL)	11 (8–14)	8 (6–11)	0.003
Total emptying fraction (%)	55 ± 7	54 ± 9	0.494
Expansion index (%)	133 (107–145)	118 (92–155)	0.353
Passive emptying fraction (%)	29 (22–34)	27 (30–34)	0.512
Passive emptying percent total emptying (%)	51 ± 14	50 ± 16	0.738
Active emptying fraction (%)	37 ± 8	37 ± 10	0.764
Booster active emptying percent total emptying (%)	49 ± 14	50 ± 16	0.738

*ASTEMI* anterior ST-elevation myocardial infarction, *LVEF* left ventricular ejection fraction, *LVEDV* left ventricular end-diastolic volume, *LVESV* left ventricular end-systolic volume, *RA* right atrium, *RVEDA* right ventricular end-diastolic area, *RVESA* right ventricular end-systolic area, *RVFAC* right ventricular fractional area change

The longitudinal 2D-STE-derived markers of RV and RA phasic functions are demonstrated in Table 3. In the patients with ASTEMI, there was a decline in RASr (33.2% ± 4.3% vs 30.5% ± 8.1%; *P* = 0.021) and RAScd (16% [12–18%] vs 14% [9–17%]; *P* = 0.048). The RV free wall late diastolic strain rate was increased in the ASTEMI group by comparison with the control group (2.2 ± 0.3 s^−1^ vs 2.6 ± 0.6 s^−1^; *P* < 0.001). The other 2D-STE-derived markers of RV and RA phasic functions were not different between the 2 study groups.

**Table 3:** Two-dimensional speckle-tracking echocardiography data of the right atrium and the right ventricle of the study groups

Variables	Control group (n = 31)	ASTEMI group (n = 92)	*P* value
RASr (%)	33.2 ± 4.3	30.5 ± 8.1	0.021
RAScd (%)	16.0 (12.0–18.0)	14.0 (9.0–17.0)	0.048
RASct (%)	17.6 ± 3.7	16.7 ± 4.9	0.359
pRASRr (s^−1^)	3.4 ± 0.4	3.4 ± 0.8	0.864
pRASRcd (s^−1^)	2.7 (2.0–3.1)	2.5 (1.8–3.1)	0.371
pRASRct (s^−1^)	4.1 ± 0.8	3.9 ± 1.1	0.405
RVFW systolic strain (%)	23.1 ± 2.6	22.7 ± 3.7	0.532
RVFW systolic strain rate (s^−1^)	2.2 ± 0.4	2.4 ± 0.5	0.057
RVFW early diastolic strain rate (s^−1^)	1.6 ± 0.4	1.8 ± 0.5	0.252
RVFW late diastolic strain rate (s^−1^)	2.2 ± 0.3	2.6 ± 0.6	< 0.001

*ASTEMI* anterior ST-elevation myocardial infarction, *pRASRcd* peak right atrial longitudinal strain rate during the conduit phase, *pRASRct* peak right atrial longitudinal strain rate during the contraction phase, *pRASRr* peak right atrial longitudinal strain rate during the reservoir phase, *RAScd* right atrial longitudinal strain during the conduit phase, *RASct* right atrial longitudinal strain during the contraction phase, *RASr* right atrial longitudinal strain during the reservoir phase, *RVFW* right ventricular free wall

The intra- and interobserver variability evaluation of the longitudinal deformation markers of the RA and the RV showed a good agreement (Additional file 1: Table S3).

There was no case of completely missed follow-ups. The follow-up duration was 16 months (95% CI 14–18 mon) with a minimum of 6 months. Four patients suffered MI, and 2 cases expired. There were no cases of stroke or hospitalization because of heart failure. The univariate Cox linear regression analysis demonstrated that RA contraction function indices were predictors of MACE occurrence (RASct: HR 0.80; 95% CI 0.67–0.97; *P* = 0.024 and pRASRct: HR 0.39; 95% CI 0.17–0.89; *P* = 0.026). The other 2DSTE-derived indices were not predictors of the occurrence of MACE (Additional file 1: Table S4).

## Discussion

In this study, we drew upon 2DSTE to evaluate RA phasic functions in patients with ASTEMI and compared the findings with those in a control group. We found that in the ASTEMI group, the reservoir and conduit functions of the RA were impaired, and maximal, minimal, and pre-P RA volume indices were reduced. In addition, RA contraction function was predictive of MACE occurrence during the follow-up period.

To our knowledge, our investigation is the first study to evaluate RA phasic functions in patients with ASTEMI. Nourian et al. [6] demonstrated that RA reservoir and conduit functions were impaired in patients with inferior MI and RVMI compared with patients with inferior MI as evaluated by 2DSTE and concluded that impairments in RA functions preceded impairments in RV function. Kanar et al. [7], in similar groups of patients, found that RA conduit function as assessed by real-time 3D echocardiography was impaired in patients with inferior MI and RVMI in comparison with patients with inferior MI but without RVMI. While their findings are similar to our findings, our study population had ASTEMI but not RVMI. Nevertheless, since some parts of the RV are supplied by the left anterior descending artery, it cannot be assumed that the RV is not damaged by ASTEMI [25].

In a study, researchers utilized color-coded tissue Doppler imaging in patients with ASTEMI and demonstrated a decline in RV systolic function [26]. They, however, failed to define the time of echocardiography after ASTEMI, and they used an echocardiography modality that was not only dependent on the ultrasound beam angle but also associated with low spatial resolution, high noise, and interobserver variability [27]. Still, it seems that echocardiography was not capable of detecting this damage to the RV in all patients with ASTEMI [28, 29]. Similar to Nourian et al., we found that RA reservoir and conduit functions were impaired in patients with ASTEMI, while we detected no significant impairment in RV function in the same phase.

In a previous study, an evaluation of LA phasic functions in patients with STEMI revealed impairments in all the functions [30]. The difference between LA and RA phasic functions in the presence of STEMI may be correlated with the difference in the pressure of the ventricle that is in interaction with the LA or the RA and the amount of damage induced by MI.

In patients with ASTEMI, the stroke volume is acutely reduced, which may lead to lower return flow to the RA [31, 32]. The lower return of blood in each cardiac cycle to the RA results in less available blood for storage during the reservoir phase and less stretching of RA myocardium. This hypothesis is supported by our finding regarding a reduced maximal RA volume index in patients with ASTEMI. These changes in the reservoir phase are followed by decreased conduit function and preserved contraction function for adaptation. The pre-P RA volume index is representative of the status of RA myocardium at precontraction. The decreased RV end-diastolic and systolic areas were in alignment with these changes. In other words, the lower return of blood to the RV and the concomitant activation of the neurohormonal axis for the compensation of the reduced cardiac output may explain the changes demonstrated in RV size and function.

According to the Frank–Starling law, the length of the myocardial fiber at precontraction is positively correlated with the amount of contraction. Further, an increased heart rate exerts a detrimental effect on myocardial contraction [33]. In patients with ASTEMI, by comparison with a control group, while RA myocardial length is reduced and heart rate is increased, contraction function is preserved, implying that the reduced myocardial length and the increased heart rate are compensated for by other factors that potentiate myocardial contraction. These factors may include changes in the sympathetic system, the renin–angiotensin–aldosterone axis, and inflammatory mediators [31, 34].

With the occurrence of acute MI, the biochemical milieu where RA myocardial fibers function changes. Such alterations lead to changes in inflammatory and neurohormonal mediators, which may affect RA phasic functions [31, 34]. Hence, the phasic functions of the RA, regardless of the volume of blood that the chamber receives, may change. Moreover, the reduced reservoir and conduit functions and the preserved contraction function imply decreased delivery of blood to the RV and ultimately to the LA and the LV. Although these changes can be regarded as an adaptation mechanism in the presence of increased filling pressure and may prevent the occurrence of pulmonary edema, in some cases, they may lead to the deterioration of LV output.

In most patients with ASTEMI, RA pressure is elevated [35]. RA phasic functions are negatively correlated with RA pressure; accordingly, increased RA pressure may result in RA phasic dysfunction through a hypothesized mechanism: impairment in the circulatory blood flow system of the RA [36]. However, increased RA pressure may be considered a marker of RV diastolic dysfunction [37].

The RA and the RV function in interaction with each other, as do the LA and the LV; thus, RA phasic changes may denote changes that occur in the RV [38]. Accordingly, impaired reservoir and conduit functions may reflect systolic and early diastolic RV dysfunction in the presence of ASTEMI.

The patterns of RA phasic functions (reduced reservoir and conduit functions and preserved contraction function) have been observed in other conditions such as increased pulmonary arterial pressure and heart failure with preserved and reduced ejection fractions [39, 40]. Nonetheless, it can be hypothesized that the continuation of RA contraction function in the presence of a reduction in the other phasic functions may lead to the exhaustion of the RA and the occurrence of atrial fibrillation [39].

Our findings regarding the prediction of MACE occurrence are not in line with those reported by Schuster et al. [5]. They applied cardiac magnetic resonance imaging to evaluate RA phasic functions and assessed the correlation between RA phasic functions and MACE. Their results demonstrated that RA conduit function was correlated with the occurrence of all-cause mortality, reinfarction, and congestive heart failure. Their sample size was larger than ours; moreover, their study population was heterogeneous and consisted of patients with non-STEMI and STEMI, including patients with RVMI. Notably, their study was a substudy of 2 other studies that utilized different vendors for cardiac magnetic resonance imaging and, thus, suffered from selection bias. In contrast, not only did we employ a single vendor for imaging and exclude patients with RVMI, but also we used echocardiography, which is more available than cardiac magnetic resonance imaging and is less costly and time-consuming.

Of all atrial phasic functions, atrial contraction function is the most independent phasic function and depends the least on ventricular function [41]. RA contraction function was preserved early after ASTEMI compared with the control group. Nevertheless, impaired RA contraction function may be a marker of adaptive mechanism failure and reflective of contraction function decompensation when other RA phasic functions are impaired.

Our study results clinically imply the potential role of RA phasic functions in the hemodynamics of patients with STEMI and could help us not only to prevent the deterioration of RA phasic functions but also to potentiate them by considering the hemodynamic status of patients. Further, our results underscore the prognostic role of RA contraction function as evaluated by 2DSTE.

### Study limitations

In the present small, single-center study, it was not feasible for us to evaluate RA phasic functions with 3D echocardiography, 3DSTE, and cardiac magnetic resonance imaging; we, therefore, assessed the phasic functions of the RA in the longitudinal direction. Another weakness of note is our use of software not originally designed for the evaluation of deformations in the RA.

## Conclusions

In our patients with ASTEMI, in comparison with a control group, the reservoir and conduit functions of the RA were reduced, while its contraction function was preserved based on 2DSTE. Additionally, no systolic and early diastolic RV dysfunction was detected, whereas there was an increase in the late diastolic RV function. In addition, RA contraction function was predictive of all-cause mortality or reinfarction.

## Supplementary Information

**Additional file 1: Table S1.** Demographic, clinical, and biochemical characteristics of the study groups. **Table S2.** Standard echocardiography data of the study groups. **Table S3.** Intra- and interobserver variabilities for 2D speckle-tracking echocardiography-derived indices of the right atrial myocardial function. **Table S4.** Two-dimensional speckle-tracking echocardiography data of the right atrium in anterior ST-elevation myocardial infarction patients with and without major adverse cardiovascular events.

## Data Availability

The data sets analyzed in the current study are available from the corresponding author on reasonable request.
