# Supplementary material for: Evaluation of the right atrial phasic functions in patients with anterior ST-elevation myocardial infarction: a 2D speckle-tracking echocardiography study
Source: BMC Cardiovasc Disord. 2022 Mar 14;22:102. doi: 10.1186/s12872-022-02546-4 (PMC8922847; doi:10.1186/s12872-022-02546-4)
Supplement: Supplementary file 1 — Additional file 1: Table S1. Demographic, clinical, and biochemical characteristics of the study groups. Table S2. Standard echocardiography data of the study groups. Table S3. Intra- and interobserver variabilities for 2D speckle-tracking echocardiography-derived indices of the right atrial myocardial function. Table S4. Two-dimensional speckle-tracking echocardiography data of the right atrium in anterior ST-elevation myocardial infarction patients with and without major adverse cardiovascular events. [file 12872_2022_2546_MOESM1_ESM.docx]

**Table 1S**: Demographic, clinical, and biochemical characteristics of the study groups

| Characteristics | Control Group (n=31) | ASTEMI Group (n=92) | P-value |
| --- | --- | --- | --- |
| Sex (male), % | 27 (87) | 80 (87) | >0.999 |
| Age, y | 55 ± 11 | 56 ± 11 | 0.636 |
| Body mass index, kg/m^2^ | 28.7 ± 3.7 | 27.8 ± 4.8 | 0.354 |
| Body surface area, m^2^ | 1.92 ± 0.20 | 1.92 ± 0.21 | 0.864 |
| Diabetes, % | 9 (29) | 27 (29) | 0.973 |
| Hypertension, % | 11 (36) | 32 (35) | 0.944 |
| Cigarette smoking, % | 7 (23) | 47 (51) | 0.006 |
| Family history of coronary artery disease, % | 6 (19) | 21 (23) | 0.686 |
| Aspirin, % | 19 (61) | 12 (13) | <0.001 |
| Statins, % | 17 (55) | 11 (12) | <0.001 |
| ACEI/ARB use, % | 7 (23) | 23 (25) | 0.786 |
| Beta-blocker use, % | 13 (42) | 7 (8) | <0.001 |
| Hemoglobin level, mg/dL | 15.2 ± 1.3 | 15.5 ± 1.6 | 0.490 |
| Fasting blood sugar, mg/dL | 101 (96 – 136) | 117 (104 – 160) | 0.014 |
| Serum triglyceride level, mg/dL | 135 (95 – 182) | 130 (98 – 204) | 0.841 |
| Serum cholesterol level, mg/dL | 156 ± 50 | 175 ± 45 | 0.054 |
| Serum high-density lipoprotein level, mg/dL | 43 ± 13 | 42 ± 9 | 0.780 |
| Serum low-density lipoprotein level, mg/dL | 81 (56 – 97) | 106 (86 – 124) | <0.001 |
| Serum creatinine level, mg/dL | 1.0 ± 0.1 | 1.0 ± 0.2 | 0.735 |

ACEI/ARB: Angiotensin-converting enzyme inhibitor/angiotensin receptor blocker; ASTEMI: Anterior ST-elevation myocardial infarction

**Table 2S**: Standard echocardiography data of the study groups

| Variables | Control Group (n=31) | ASTEMI Group (n=92) | P-value |
| --- | --- | --- | --- |
| Heart Rate, bpm | 62 ± 9 | 79 ± 13 | <0.001 |
| Systolic blood pressure, mm Hg | 126 ± 15 | 120 ± 17 | 0.117 |
| Diastolic blood pressure, mm Hg | 80 ± 7 | 78 ± 11 | 0.310 |
| LVEDV index, mL/m^2^ | 49 ± 9 | 50 ± 11 | 0.542 |
| LVESV index, mL/m^2^ | 20 ± 4 | 29 ± 8 | <0.001 |
| LVEF,% | 59 ± 6 | 42 ± 7 | <0.001 |
| RV diameter, mm | 30 ± 3 | 29 ± 7 | 0.199 |
| TAPSE, mm | 23 ± 3 | 20 ± 7 | 0.063 |
| RVEDA, cm^2^ | 19.7 ± 3.3 | 16.5 ± 4.6 | 0.001 |
| RVESA, cm^2^ | 10.8 ± 2.2 | 8.1 ± 2.9 | <0.001 |
| RVFAC, % | 45 ± 7 | 51 ± 9 | 0.001 |
| Tricuspid E, cm/s | 49 ± 9 | 48 ± 11 | 0.736 |
| Tricuspid A, cm/s | 40 ± 9 | 41 ± 10 | 0.689 |
| Tricuspid DT, msec | 216 ± 46 | 194 ± 45 | 0.021 |
| Tricuspid E/A ratio | 1.3 ± 0.3 | 1.2 ± 0.3 | 0.544 |
| Systolic pulmonary artery pressure, mm Hg* | 23 ± 4 | 28 ± 4 | 0.063 |
| RV s′, cm/s | 11.4 ± 2.0 | 11.9 ± 2.0 | 0.204 |
| RV e′, cm/s | 8.1 ± 2.0 | 8.6 ± 2.2 | 0.307 |
| RV a′, cm/s | 12.6 ± 2.9 | 15.7 ± 3.9 | <0.001 |
| RV E/e′ ratio | 6.3 ± 1.8 | 5.9 ± 2.1 | 0.407 |
| RV e′/a′ ratio | 0.7 ± 0.2 | 0.6 ± 0.2 | 0.024 |
| Maximum RA volume index, mL/m^2^ | 20 (17 – 27) | 17 (13 – 22) | <0.001 |
| Minimum RA volume index, mL/m^2^ | 9 (7 – 13) | 7 (6 – 10) | 0.005 |
| Pre-P RA volume index, mL/m^2^ | 15 (13 – 21) | 12 (10 – 15) | <0.001 |
| Total emptying volume, mL | 21 (20 – 29) | 17 (12 – 23) | 0.001 |
| Passive emptying volume, mL | 11 (9 – 14) | 8 (5 – 13) | 0.005 |
| Active emptying volume, mL | 11 (8 – 14) | 8 (6 – 11) | 0.003 |
| Total emptying fraction, % | 55 ± 7 | 54 ± 9 | 0.494 |
| Expansion index,% | 133 (107 – 145) | 118 (92 – 155) | 0.353 |
| Passive emptying fraction, % | 29 (22 – 34) | 27 (30 – 34) | 0.512 |
| Passive emptying percent total emptying, % | 51 ± 14 | 50 ± 16 | 0.738 |
| Active emptying fraction, % | 37 ± 8 | 37 ± 10 | 0.764 |
| Booster active emptying percent total emptying, % | 49 ± 14 | 50 ± 16 | 0.738 |

* Systolic pulmonary artery pressure was measurable in 17 subjects in the control group and 66 patients in the ASTEMI group.

A: Peak velocity of the tricuspid flow in late diastole, a′: Peak velocity of late diastolic motion, ASTEMI: Anterior ST-elevation myocardial infarction, DT: Deceleration time, E: Peak velocity of the tricuspid flow in early diastole, e′: Peak velocity of the early diastolic motion, LVEF: Left ventricular ejection fraction, LVEDV: Left ventricular end-diastolic volume index, LVESV: Left ventricular end-systolic volume index, RA: Right atrium, RVEDA: Right ventricular end-diastolic area, RVESA: Right ventricular end-systolic area, RVFAC: Right ventricular fractional area change, RVFW: Right ventricular free wall, s′: Peak velocity of the systolic motion, TAPSE: Tricuspid annular plane systolic excursion

**Table 3S**: Intra- and interobserver variabilities for 2D speckle-tracking echocardiography-derived indices of the right atrial myocardial function

| Variable | Intraobserver | | Interobserver | |
| --- | --- | --- | --- | --- |
|  | ICC | 95% limit of agreement | ICC | 95% limit of agreement |
| RASr ,% | 0.982 | 0.962 – 0.992 | 0.968 | 0.930 – 0.985 |
| RAScd ,% | 0.993 | 0.985 – 0.997 | 0.976 | 0.647 – 0.989 |
| RASct ,% | 0.973 | 0.941 – 0.988 | 0.956 | 0.903 – 0.980 |
| pRASRr, s^-1^ | 0.851 | 0.677 – 0.932 | 0.868 | 0.712 – 0.940 |
| PRASRcd, s^-1^ | 0.983 | 0.963 – 0.992 | 0.952 | 0.896– 0.978 |
| pRASRct , s^-1^ | 0.958 | 0.909 – 0.981 | 0.881 | 0.739 – 0.941 |

ICC, Intraclass correlation coefficient; pRASRcd, Peak right atrial longitudinal strain rate during the conduit phase; pRASRct, Peak right atrial longitudinal strain rate during the contraction phase; pRASRr, Peak right atrial longitudinal strain rate during the reservoir phase; RAScd, Right atrial longitudinal strain during the conduit phase; RASct, Right atrial longitudinal strain during the contraction phase; RASr, Right atrial longitudinal strain during the reservoir phase

**Table 4S**: Two-dimensional speckle-tracking echocardiography data of the right atrium in anterior ST-elevation myocardial infarction patients with and without major adverse cardiovascular events.

| Group  Variables | Patients without MACE  (n=86) | Patients with MACE  (n=6) | HR  (95% CI) | P-value |
| --- | --- | --- | --- | --- |
| RASr ,% | 30.5 (26.0 - 35.9) | 24.0 (16.5 - 40.6) | 0.92 (0.83-1.03) | 0.143 |
| RAScd ,% | 13.7 (9.2 - 16.9) | 13.5 (5.3 - 23.1) | 0.99 (0.87-1.13) | 0.894 |
| RASct ,% | 16.7 (13.9 - 19.8) | 11.3 (10.3 - 15.8) | 0.80 (0.67-0.97) | 0.024 |
| pRASRr, s^-1^ | 3.4 (2.9 - 4.1) | 3.1 (2.2 - 4.0) | 0.58 (0.23-1.48) | 0.253 |
| PRASRcd, s^-1^ | 2.5 (1.8 - 3.0) | 2.4 (1.3 - 4.8) | 1.29 (0.55-3.02) | 0.563 |
| pRASRct , s^-1^ | 4.1 (3.1 - 4.7) | 2.9 (2.4 - 3.8) | 0.39 (0.17-0.89) | 0.026 |

CI, Confidence interval; MACE, Major adverse cardiovascular events; pRASRcd, Peak right atrial longitudinal strain rate during the conduit phase; pRASRct, Peak right atrial longitudinal strain rate during the contraction phase; pRASRr, Peak right atrial longitudinal strain rate during the reservoir phase; RAScd, Right atrial longitudinal strain during the conduit phase; RASct, Right atrial longitudinal strain during the contraction phase; RASr, Right atrial longitudinal strain during the reservoir phase
